# Adenosine A_2A_ and dopamine D_2_ receptor interaction controls fatigue resistance

**DOI:** 10.3389/fphar.2024.1390187

**Published:** 2024-05-27

**Authors:** Ana Cristina de Bem Alves, Naiara de Souza Santos, Ana Paula Tavares Santos, Gabriela da Panatta, Ana Elisa Speck, Rodrigo A. Cunha, Aderbal S. Aguiar

**Affiliations:** ^1^ Biology of Exercise Lab, Department of Health Sciences, UFSC-Federal University of Santa Catarina, Araranguá, Brazil; ^2^ CNC-Center for Neuroscience and Cell Biology, University of Coimbra, Coimbra, Portugal; ^3^ FMUC—Faculty of Medicine, University of Coimbra, Coimbra, Portugal

**Keywords:** caffeine, central fatigue, DPCPX, haloperidol, SCH58261, striatum

## Abstract

**Introduction:** Caffeine and the selective A_2A_ receptor antagonist SCH58261 both have ergogenic properties, effectively reducing fatigue and enhancing exercise capacity. This study investigates in male Swiss mice the interaction between adenosine A_2A_ receptors and dopamine D_2_ receptors controlling central fatigue, with a focus on the striatum where these receptors are most abundant.

**Methods:** We employed DPCPX and SCH58261 to antagonize A_1_ and A_2A_ receptors, caffeine as a non-competitive antagonist for both receptors, and haloperidol as a D_2_ receptor antagonist; all compounds were tested upon systemic application and caffeine and SCH58261 were also directly applied in the striatum. Behavioral assessments using the open field, grip strength, and treadmill tests allowed estimating the effect of treatments on fatigue.

**Results and discussion:** The results suggested a complex interplay between the dopamine and adenosine systems. While systemic DPCPX had little effect on motor performance or fatigue, the application of either caffeine or SCH58261 was ergogenic, and these effects were attenuated by haloperidol. The intra-striatal administration of caffeine or SCH58261 was also ergogenic, but these effects were unaffected by haloperidol. These findings confirm a role of striatal A_2A_ receptors in the control of central fatigue but suggest that the D_2_ receptor-mediated control of the ergogenic effects of caffeine and of A_2A_ receptor antagonists might occur outside the striatum. This prompts the need of additional efforts to unveil the role of different brain regions in the control of fatigue.

## 1 Introduction

Central fatigue, characterized by a reduced ability to maintain cognitive and physical performance, stems from intricate neurobiological (Gandevia, 2001; Davis et al., 2003; Meeusen et al., 2006; Aguiar et al., 2020) and metabolic interactions (Glaister, 2005; Wan et al., 2017). Studies linking aerobic performance, oxidative metabolism, and fatigue have shown that blocking brain adenosine receptors increases resistance to physical fatigue (Davis et al., 2003; Aguiar et al., 2020), highlighting the significant role of adenosine in fatigue signaling (Aguiar et al., 2021). Moreover, by integrating pharmacological research with knockout mouse models, we have previously demonstrated that adenosine A_2A_ receptors (A_2A_R) are crucial for the performance-enhancing effects of caffeine (Aguiar et al., 2020). However, non-toxic doses of caffeine selectively antagonize both adenosine A_2A_ and A_1_ receptors to format information flow within brain neuronal circuits (Lopes et al., 2019) and it remains to be clarified if A_1_R also play a role in controlling fatigue.

Additionally, we identified a specific role of A_2A_R in the striatum for controlling central fatigue (de Bem Alves et al., 2023). Notably, A_2A_R interacts closely with dopamine D_2_ receptors (D2R) in this region, forming A_2A_R-D_2_R heteromers (Ferré et al., 2008; Ferré and Ciruela, 2019), a concept pioneered by Kell Fuxe in the early 90’s (Ferré et al., 1991). These interactions are significant in various conditions, including Parkinson’s disease, schizophrenia, substance abuse, and Attention Deficit Hyperactivity Disorder (ADHD), as reviewed in the literature (Fuxe et al., 2003; Ballesteros-Yáñez et al., 2018; Chen et al., 2023). Consequently, the antagonism of D_2_R by haloperidol affects the efficacy of A_2A_R antagonists in modulating effort-related behaviors (Salamone et al., 2009; Pardo et al., 2012; Rotolo et al., 2023). While A_2A_R-D_2_R interactions have been observed in other areas of the brain (Pandolfo et al., 2013; Dremencov et al., 2017; Real et al., 2018), their functional significance remains less understood.

This study aimed to evaluate the potential ergogenic effects of selective antagonists for A_1_R and A_2A_R, along with the non-selective adenosine receptor antagonist caffeine. Our objective was to determine whether and how these effects are altered by a D_2_R antagonist, particularly in the striatum, an area previously shown to influence central fatigue (Davis et al., 2003; Aguiar et al., 2020; de Bem Alves et al., 2023). Additionally, we venture into new territory by examining the impact of adenosine receptors on exercise physiology, specifically focusing on strength performance, which is predominantly dependent on anaerobic metabolism. This approach allows us to expand our understanding of fatigue mechanisms across various physical activities and metabolic requirements.

## 2 Methods

### 2.1 Animals

We used one hundred and twelve (112) male Swiss mice (49.4 ± 1.5 g, 8–10 weeks old), housed in collective cages (38 × 32 × 17 cm) maintained on a 12-h light-dark cycle at a controlled room temperature of 22°C ± 1°C, and food and water *ad libitum*. The sample size for ANOVA comparison was set at α = 0.05 and β = 0.8. The experimental protocol (CEUA 1503210519) was granted approval by the Animal Care and Use Committee (IACUC) of the Universidade Federal de Santa Catarina (UFSC). The assignment of mice to experimental groups was random, with each animal treated as an individual experimental unit for every test.

### 2.2 Drugs

The first experiments were the systemic treatments. The drug administration schedule was as follows: caffeine at 6.0 mg/kg and SCH58261 at 1.0 mg/kg were administered 15 min before the behavioral tests while haloperidol (Haldol^®^) at 250 μg/kg and DPCPX at 1.0 mg/kg were administered 30 min prior. The dosages and administration times for caffeine (Solano et al., 2017; Aguiar et al., 2020; de Bem Alves et al., 2023), SCH58261 (El Yacoubi et al., 2000a; Aguiar et al., 2020), and DPCPX (Griebel et al., 1991; El Yacoubi et al., 2000b; Prediger et al., 2004; Li et al., 2018; Szopa et al., 2018; 2021) were determined based on prior pilot studies and existing evidence. Doses of 0.25–1.0 mg/kg, i.p. of haloperidol were tested in the catalepsy test (Shiozaki et al., 1999; Mihara et al., 2007), showing that 250 μg/kg did not cause either catalepsy or motor impairment (see Results). Caffeine and haloperidol were diluted in saline (0.9% NaCl) whereas SCH58261 and DPCPX were diluted in 5.0% DMSO. Control groups received either saline or DMSO vehicles. The systemic treatment was intraperitoneal (i.p.) at a volume of 10 mL/kg. All drugs were purchased from Sigma-Aldrich.

For stereotactic surgeries, mice were anesthetized with ketamine/xylazine for cannula implantation in both the right (AP 0.5 mm; ML 2.0 mm; DV −3.0 mm) and left (AP 0.5 mm; ML −2.0 mm; DV −3.0 mm) striata, using Paxinos and Franklin’s stereotaxic coordinates (Paxinos and Franklin, 2012). One-week post-surgery, we injected 4 μL at 2 μL/min of either saline, DMSO, caffeine (15 μg) or SCH58261 (2 μg) into the conscious animals immediately before the behavioral tests. Haloperidol (250 μg/kg, i.p) was given 30 min before the behavioral tests. The mortality rate was 5% (10 animals). The placement of the cannulae was verified post-euthanasia by dissecting the mouse brain.

### 2.3 Motor and fatigue behavioral tests

Experiments were conducted from 9 a.m. to 5 p.m. during the mice’s light circadian phase in a sound-controlled room, maintaining controlled temperature, humidity, and low-intensity light (approximately 10 lx). The behavioral apparatus was cleaned with 10% ethanol after each trial to ensure cleanliness and prevent contamination. Additionally, the order of the tests was randomized to eliminate bias.

[Open field] Each mouse was allowed to explore a circular arena with a diameter of 600 mm for 5 min using the Insight^®^ EP154 apparatus (Ribeirão Preto, SP, Brazil). A manual count of the number of crossings was then carried out.
[Grip strength test] We used the Bonther^®^ 5 kgf grip strength meter (Bonther, Ribeirão Preto, SP, Brazil) to evaluate fatigue, placing each mouse on the bar and gently pulling its tail, opposing the firm grip of its front paws over four 10-s trials (de Bem Alves and Aguiar, 2024). The final score is the average strength measured in three trials (Personius et al., 2010; Takeshita et al., 2017).
[Treadmill incremental running test] Mice were habituated to a mouse treadmill (Bonther, Ribeirão Preto, SP, Brazil) over 3 days. The regimen started with a 10-min session at 5 m/min, followed by a 5-min session each at 5 m/min and 10 m/min, and concluded with 10 min at 10 m/min. After a 48-h rest period, they underwent an incremental test where the belt speed increased by 5 m/min every 3 min, conducted at a 1.7° slope and with a 0.2 mA shock intensity, to estimate (vertical) running power (AS Aguiar et al., 2018; Aguiar et al., 2020). The external length of the cannulas limited treadmill testing to only those animals treated systemically.

### 2.4 Statistical analysis

Data are presented as means ± SEM, generated using GraphPad Prism version 10 (GraphPad Software, San Diego, California, United States; www.graphpad.com). Adhering to the intention-to-treat principle, statistical analysis was conducted using STATISTICA version 13.5.0.17 (StatSoft, Inc.; www.statsoft.com). The analyses included one-way ANOVA with Tukey’s *post hoc* test and repeated measures ANOVA with Bonferroni’s *post hoc* test for fatigue resistance (force × time). Effect sizes were evaluated using Cohen’s η^2^, categorizing them as small (0.01), medium (0.09), or large (0.25). Statistical power (β) was also assessed. An alpha level of *p* < 0.05 was used to determine significance.

### 2.5 Data availability statement

This work is available as open data under the Creative Commons Attribution (CC BY) license. For details, see (Speck et al., 2024).

## 3 Results

**FIGURE 1:**
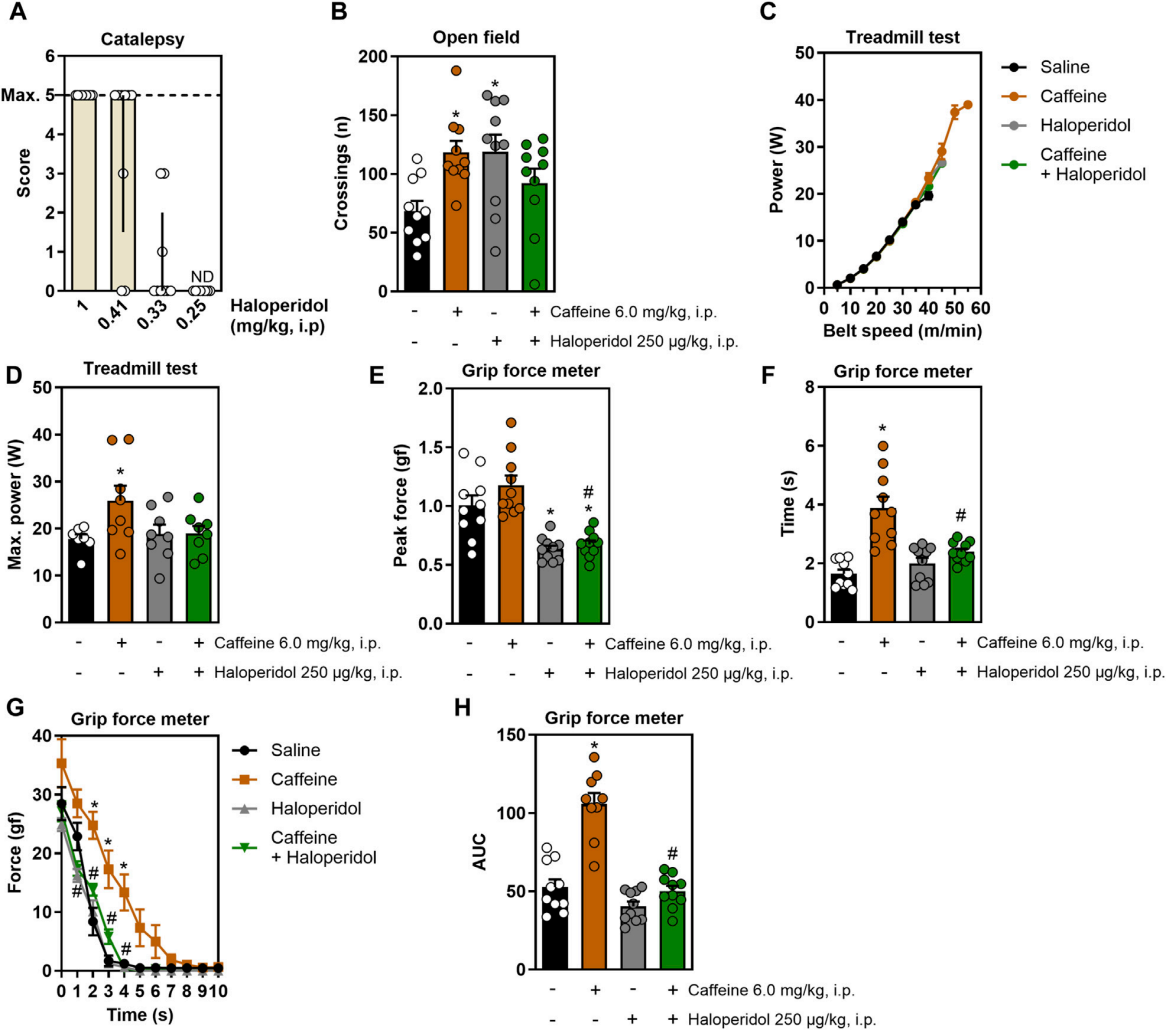
Effects of systemic treatment with caffeine and/or haloperidol on locomotion, running power and grip strength. Panel **(A)** shows the dose-response curve for haloperidol in the catalepsy test, identifying 250 μg/kg as a non-cataleptic dose. Panel **(B)** shows that systemic administration of caffeine increased the number of crossings in the open field, which is reduced by a non-cataleptic dose of haloperidol. Panel **(C,D)** show that systemic caffeine enhances running power in the incremental treadmill test, an effect prevented by haloperidol. Panel **(E–G)** show that systemic caffeine improved grip time **(F)** and impulse **(H)**, without changes in peak strength **(E)**, and improved the resistance to fatigue **(G)**, all these effects being attenuated by haloperidol. Results are shown as median ± interquartile range **(A)** or mean ± standard error of the mean **(B–H)**. N = 8–10 animals/group for 2-3 independent experiments. Statistical significance (*p* < 0.05) *versus* control (*) or *versus* caffeine (#), with effect sizes (η^2^) and power (β) confirming robust results, was determined using ANOVA and *post hoc* tests, as detailed in the Methods’ section. AUC, area under the curve. ND, not detectable.

Figure 1A displays the dose-response curve of haloperidol in the catalepsy test, which prompted selecting the non-cataleptic dose of 250 μg/kg. As expected, systemic caffeine acted both as a psychostimulant and as an ergogenic agent: it increased open field crossings, an effect mitigated by the non-cataleptic dose of haloperidol (F_3,35_ = 4.3, η^2^ = 0.27, β = 0.83, *p* < 0.05, Figure 1B). In the incremental treadmill test (Figure 1C), caffeine boosted running power, an effect that was not affected by haloperidol (F_3,27_ = 3.2, η^2^ = 0.25, β = 0.67, *p* < 0.05, Figure 1D). In the grip strength test, caffeine enhanced grip time (F_3,35_ = 17.5, η^2^ = 0.6, β = 0.99, *p* < 0.05, Figure 1F) and impulse (F_3,34_ = 36.4, η^2^ = 0.76, β = 1.0, *p* < 0.05, Figure 1H) without increasing peak strength (Figure 1E), and haloperidol mitigated these effects. Additionally, caffeine improved the resistance to fatigue in the grip test, a benefit not present in animals also receiving haloperidol (F_30,350_ = 4.7, η^2^ = 0.29, β = 1.0, *p* < 0.05, Figure 1G).

**FIGURE 2:**
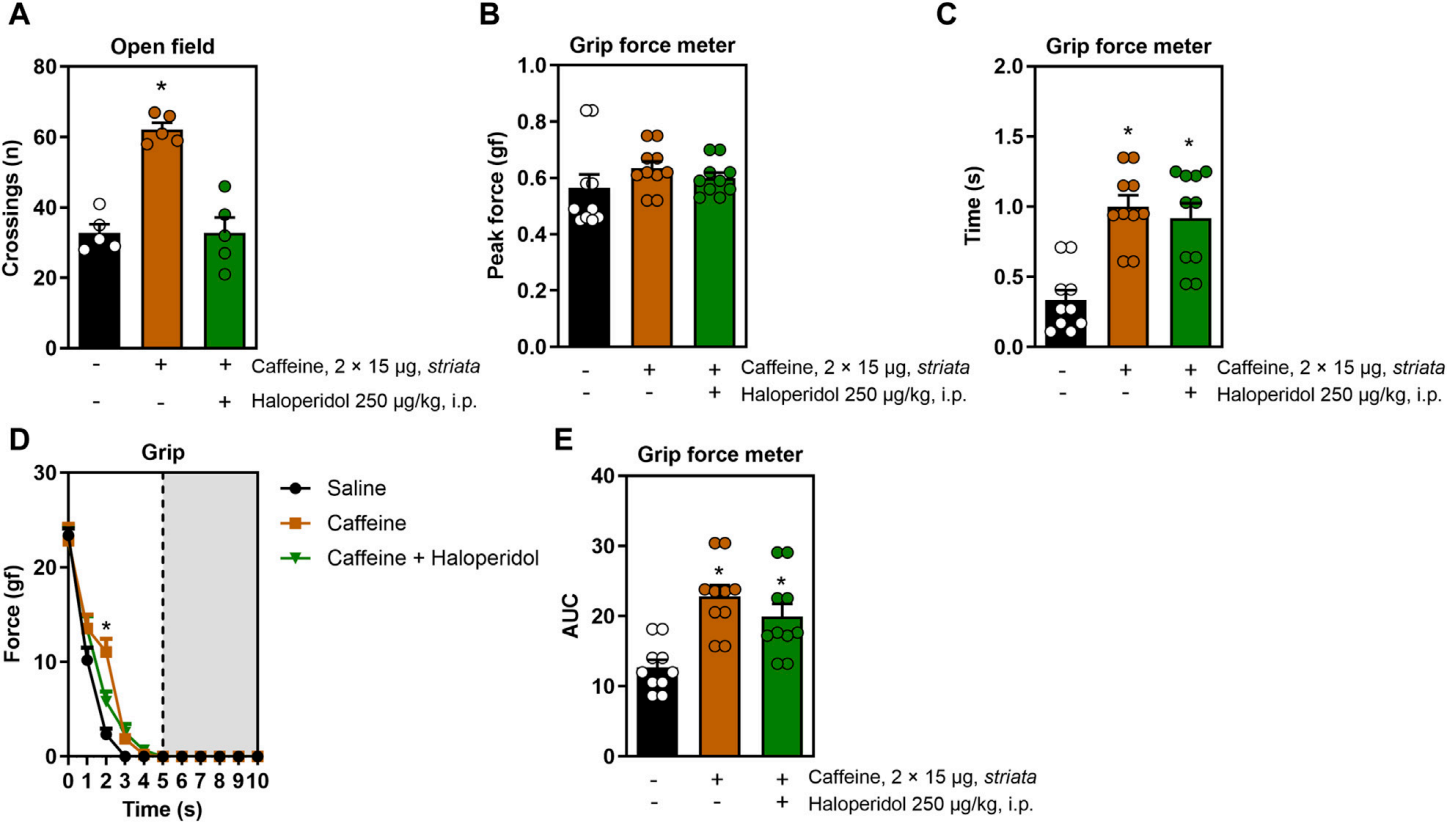
Effects of intra-striatal injection with caffeine, without or with systemic administration of haloperidol, on locomotion and grip strength. Panel **(A)** shows that caffeine increased the number of open field crossings, an effect mitigated by haloperidol. Panel **(B)** shows a lack of modification of the peak grip strength upon treatments with intra-striatal caffeine without or with systemic haloperidol. Intra-striatal caffeine improved grip time **(C)**, fatigue resistance **(D)** and impulse **(E)** and all these effects were not significantly modified by systemic treatment with haloperidol. Data are mean ± standard error of the mean. N = 8–10 animals/group for 2-3 independent experiments. Statistical significance (*p* < 0.05) *versus* control (*) or *versus* caffeine (#), was confirmed by effect sizes (η^2^) and power (β) through ANOVA and *post hoc* analysis, as elaborated in the Methods’ section. AUC, area under the curve.

When applied directly in the striatum, caffeine also triggered a psychostimulant and ergogenic response: it increased the number of open field crossings (F_2,12_ = 31.1, η^2^ = 0.83, β = 0.99, *p* < 0.05, Figure 2A), grip time (F_2,26_ = 6.4, η^2^ = 0.055, β = 0.99, *p* < 0.05, Figure 2C), resistance to fatigue (F_8,104_ = 4.0, η^2^ = 0.23, β = 0.98, *p* < 0.05, Figure 2D) and impulse (F_2,12_ = 31.1, η^2^ = 0.83, β = 0.99, *p* < 0.05, Figure 2E) without increasing peak strength (Figure 2B). Strikingly, although haloperidol mitigated the psychostimulant effect of caffeine in the open field, it did not alter the ergogenic properties of intra-striatal caffeine in the treadmill and grip tests.

**FIGURE 3:**
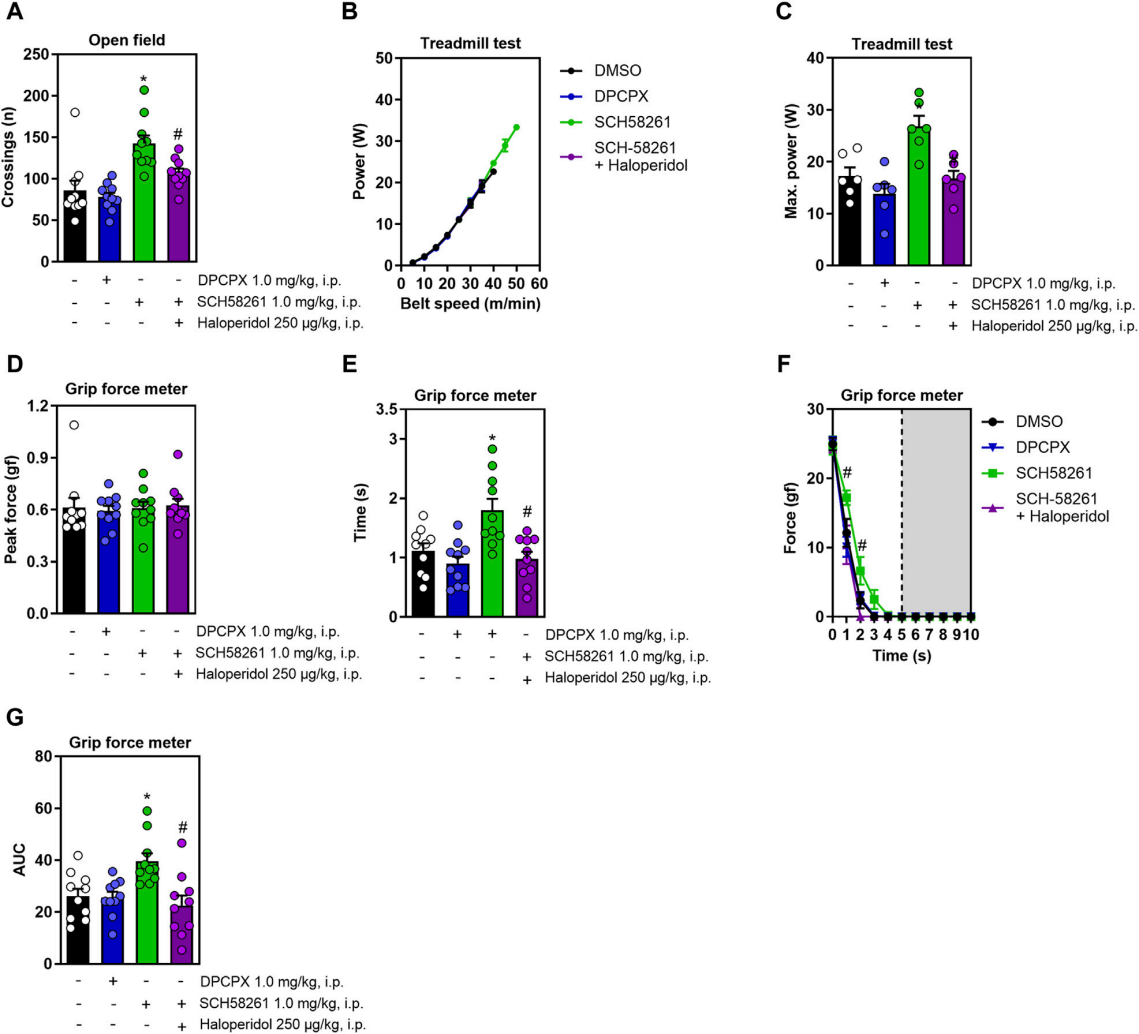
Effects on locomotion, running power and grip strength of systemic treatment with DPCPX or with SCH58261 without or with haloperidol. DPCPX was devoid of effects in all behavioral measures. Panel **(A)** shows that systemic SCH58261 increased locomotion in the open field, an effect abrogated by haloperidol. Panel **(B,C)** show that systemic SCH58261 increased running power, an effect prevented by haloperidol. In the grip test, systemic SCH58261 did not modify peak grip strength **(D)** but prolonged grip time **(E)**, increased resistance to fatigue **(F)** and endurance (AUC, Figure 3G), all these effects being prevented by haloperidol. N = 8–10 animals/group for 2-3 independent experiments. Statistical significance (*p* < 0.05) *versus* control (*) or *versus* SCH58261 (#), was confirmed by effect sizes (η^2^) and power (β) through ANOVA and *post hoc* analysis, as elaborated in the Methods’ section. AUC, area under the curve.

Since caffeine non-competitively antagonizes adenosine A_1_ and A_2A_ receptors (Fredholm et al., 1999), we exposed animals to DPCPX and SCH58261, which are selective antagonists for these respective receptors. DPCPX administration did not alter any measured behaviors, whereas SCH58261 increased locomotion in the open field; this effect was nullified in the presence of haloperidol (F_3,36_ = 11.4, η^2^ = 0.48, β = 0.99, *p* < 0.05, Figure 3A). Figure 3B shows the increase in running power with increased speed. SCH58261 also significantly improved running power in the treadmill test, a gain blocked by haloperidol (F_3,20_ = 9.8, η^2^ = 0.59, β = 0.99, *p* < 0.05, Figure 3C). While treatments did not affect peak grip strength (Figure 3D), SCH58261 enhanced both grip time (F_3,36_ = 8.3, η^2^ = 0.41, β = 0.98, *p* < 0.05, Figure 3E) and area under the curve (AUC, impulse) (F_3,36_ = 6.3, η^2^ = 0.34, β = 0.94, *p* < 0.05, Figure 3G), with haloperidol diminishing these effects. Additionally, haloperidol reduced resistance to fatigue in SCH58261-treated animals (F_12,144_ = 1.9, η^2^ = 0.14, β = 0.9, *p* < 0.05, Figure 3F).

**FIGURE 4:**
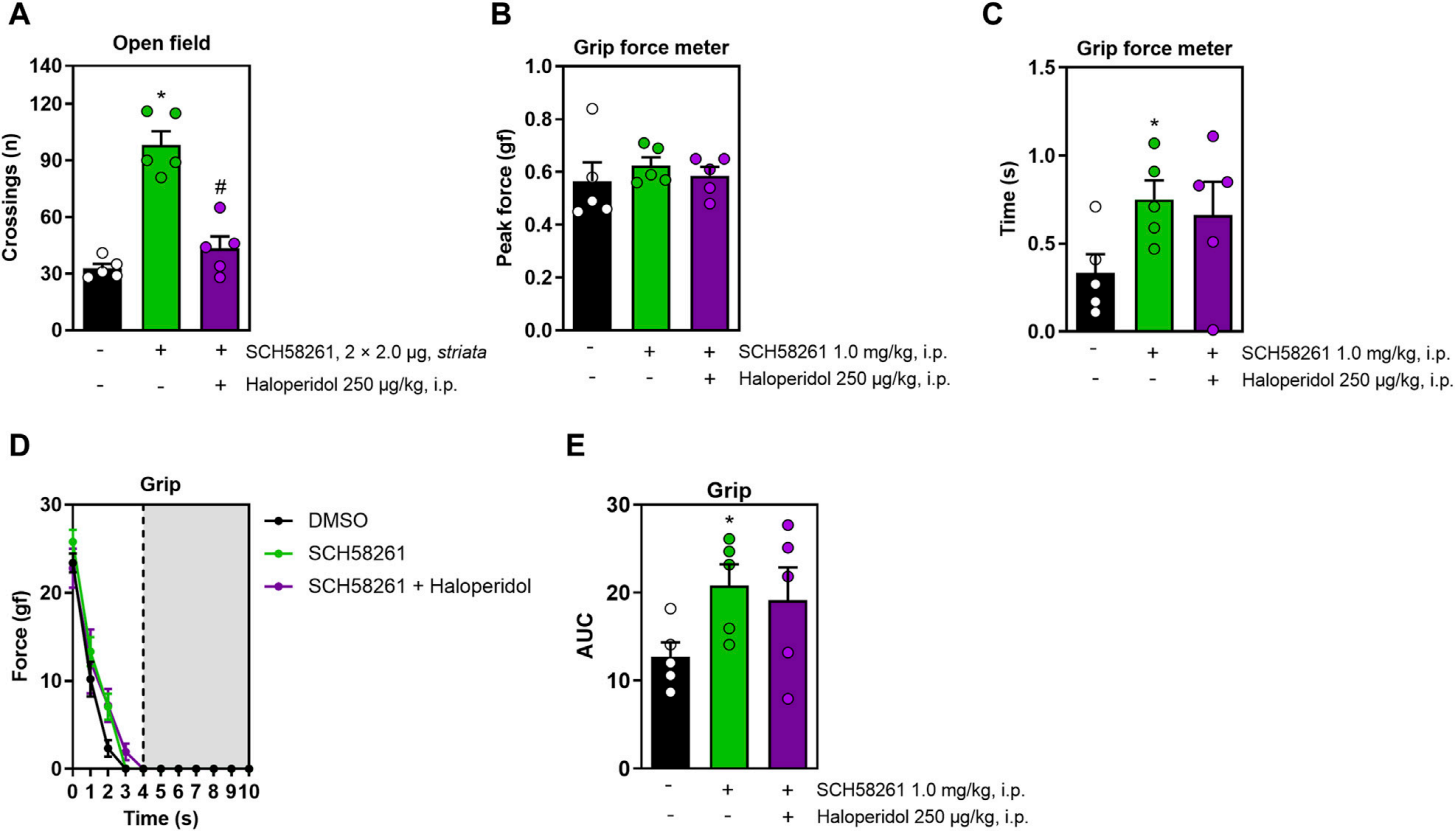
Effects of the intra-striatal injection of SCH58261 without or with systemic haloperidol on locomotion and grip strength. Panel **(A)** shows that intra-striatal SCH58261 increased locomotion in the open field test, an effect attenuated by haloperidol. Panel **(B)** confirmed that intra-striatal SCH58261 did not alter peak grip strength but increased grip time **(C)** and endurance (AUC, Figure 4E), both effects unaffected by haloperidol. Panel **(D)** shows a consistent strength decline, unaffected by any of the treatments. Data are mean ± standard error of the mean. N = 5 animals/group for 2 independent experiments. Significance (*p* < 0.05) *versus* control (*) or *versus* SCH58261 (#), supported by effect sizes (η^2^) and power (β), is based on ANOVA and *post hoc* analysis, as explained in the Methods’ section. AUC, area under the curve.

Lastly, we assessed the intra-striatal effects of SCH58261, foregoing DPCPX due to its lack of systemic effects. Intra-striatal administration of SCH58261 increased locomotion in the open field, an effect that was mitigated by haloperidol (F_2,12_ = 37.7, η^2^ = 0.86, β = 1.00, *p* < 0.05, Figure 4A). While intra-striatal SCH58261 did not affect peak grip strength (Figure 4B), it prolonged grip time (F_2,27_ = 5.5, η^2^ = 0.29, β = 0.80, *p* < 0.05, Figure 4C) and enhanced impulse (F_2,27_ = 5.5, η^2^ = 0.28, β = 0.80, *p* < 0.05, Figure 4E). All animals exhibited a decline in strength over time, with no significant differences between treatments observed (F_6,81_ = 2.7, η^2^ = 0.16, β = 0.84, *p* < 0.05, Figure 4D). Notably, alterations induced by intra-striatal SCH58261 in grip strength were not modified by haloperidol.

All effect sizes were large, except for the resistance to fatigue comparisons following systemic treatment with DPCPX or SCH58261 (Figure 3F), and intra-striatal treatment with caffeine (Figure 2C), which exhibited medium effect sizes. Generally, the statistical power for our analyses was high, exceeding 80%. The only exception was the systemic treatment with caffeine and haloperidol (Figure 1D), which had a statistical power of 67%; however, this did not significantly impact our overall conclusions.

## 4 Discussion

The present study confirmed that the systemic administration of either caffeine and of the selective antagonist of A_2A_R, SCH58261 were ergogenic and that these effects were largely reproduced by the direct injection of caffeine or SCH58261 in the striatum, aligning with previous findings (de Bem Alves et al., 2023) and the general role of striatal A_2A_R in effort-related behaviors, reviewed in Chen et al. (2023).

The first novelty provided by this study is the re-enforcement of our previous contention that A_2A_R are the likely molecular targets operated by caffeine to produce its ergogenic effects (Aguiar et al., 2020). Physiological concentrations of caffeine, achieved in the brain parenchyma after consuming non-toxic doses, influence neuronal network information flow primarily through A_1_R and A_2A_R antagonism (Lopes et al., 2019), without involving other mechanisms such as phosphodiesterases or ryanodine receptors, which are activated by toxic caffeine doses (Fredholm et al., 1999). Our findings that caffeine’s ergogenic effects are replicated by the selective A_2A_R antagonist SCH58261, but not by the A_1_R antagonist DPCPX, provide direct evidence that caffeine’s ergogenic effects are specifically mediated through A_2A_R rather than A_1_R. Additionally, caffeine’s influence on other behavioral responses that rely on striatal information processing, such as locomotion, arousal, or response to psychostimulants, is also mediated by A_2A_R rather than A_1_R (El Yacoubi et al., 2000a; Chen et al., 2000). Therefore, the specific involvement of A_2A_R, rather than A_1_R, observed in the acute effects of caffeine on exercise performance, further supports the significant role of striatal A_2A_R in governing caffeine’s ergogenic effects (Aguiar et al., 2020). This is especially pertinent given the high density of A_2A_R in the striatum compared to other brain regions, which predominantly exhibit A_1_R-mediated effects following acute caffeine exposure (Elmenhorst et al., 2012; Kerkhofs et al., 2018).

Another significant conclusion drawn from this study pertains to the interaction between A_2A_R and D_2_R in managing exercise endurance. This presumed interplay between A_2A_R and D_2_R is supported by: i) their co-localization within medium spiny neurons of the striatum (Svenningsson et al., 1997; Canals et al., 2003), a region recognized for the ergogenic effects prompted by caffeine blockade (de Bem Alves et al., 2023); ii) their established functional crosstalk (Hillion et al., 2002; Ferré et al., 2016); iii) their dynamic heteromerization, which results in new receptosomes with novel properties (Canals et al., 2003; Fernández-Dueñas et al., 2015); iv) their interplay in controlling motor activity, the impact of psychostimulants, and effort-related behavioral performance (e.g., Pardo et al., 2013; reviewed in Borroto-Escuela et al., 2018; Nunes et al., 2010; Rotolo et al., 2023). Additionally, dopamine signaling has been experimentally linked to exercise and fatigue (reviewed in Meeusen et al., 2021). Exercise induces a hyperdopaminergic state (Greenwood, 2019) and dopamine depletion correlates with mental fatigue (Moeller et al., 2012; Aguiar et al., 2016; Scheffer et al., 2021). Furthermore, polymorphisms within various components of the dopaminergic system have been associated with fatigue (Malyuchenko et al., 2010), L-DOPA has been shown to alleviate physical fatigue (Lou et al., 2003; Scheffer et al., 2021; de Bem Alves and Aguiar, 2024), and exposure to reserpine, which depletes monoamines, serves as an experimental fatigue model (Scheffer et al., 2021; de Bem Alves and Aguiar, 2024).

Despite these findings, the specific dopamine receptors involved in the control of fatigue and their brain locations remain unclear. The traditional view of striatal dopaminergic signaling suggests a primary role for D_1_R, but the simplistic dichotomy of behaviors influenced by D_1_R and D_2_R in medium spiny neurons is being reevaluated (Soares-Cunha et al., 2016). The improvement of performance with increased forced running is linked to dopamine and specifically to the activation of striatal D_1_R and extrastriatal D_2_R (Toval et al., 2021). Additionally, the dorsolateral prefrontal cortex has been proposed as important during exhaustive exercise (Bigliassi and Filho, 2022). These insights align with our unexpected findings: i) haloperidol reduces the ergogenic effects of systemically administered caffeine or SCH58261; ii) notably, haloperidol does not affect the ergogenic effects when caffeine or SCH58261 is applied directly to the striatum.

These results lead us to conclude that A_2A_R-D_2_R interactions play a significant role in regulating exercise endurance and fatigue, predominantly outside the striatum. This suggests that adenosine’s central role in exercise endurance and fatigue might involve primarily changes in striatal circuits, but also modifications in other brain areas. This concept aligns with the noted involvement of striatal and extrastriatal circuits in other behaviors affected by A_2A_R-D_2_R interplay, such as responses to psychostimulants (reviewed in Borroto-Escuela et al., 2018; Chen et al., 2023). However, this conclusion that the extra-striatal A_2A_R-D_2_R interplay is involved in ergogenic effects should still be regarded as preliminary since it will require confirmation based on direct pharmacological and genetic manipulations of A_2A_R and D_2_R only in the striatum and the identification of the brain regions where this interaction takes place to control fatigue. Additionally, future studies should also investigate a putative role of dopamine D_1_R, which is also abundantly present in the striatum and other brain regions and has also been reported to tightly interact with adenosine A_1_R (Ginés et al., 2000) and dopamine D_2_R (Rashid et al., 2007; Frederick et al., 2015).

Interestingly, our current findings indicate that the ergogenic effects of caffeine and SCH58261 are distinct from their psychostimulant properties. This observation is supported by our previous research showing that SCH58261 has both psychostimulant and ergogenic effects in male mice, but only ergogenic effects in females, without clear psychostimulant impacts (Aguiar et al., 2020). This adds to the growing body of evidence suggesting a unique role for various A_2A_R populations in different cellular locations in modulating diverse behavioral responses (Shen et al., 2008; Yu et al., 2008; Wei et al., 2014).

In summary, our study confirms the critical role of striatal A_2A_R in the adenosine modulation of central fatigue and in the ergogenic responses of A_2A_R antagonists. Furthermore, we found a dissociation between the psychostimulant and ergogenic effects of caffeine and SCH58261. In parallel, the ergogenic effects of caffeine and of SCH568261 were controlled by D_2_R blockade with haloperidol, but this A_2A_R-D_2_R interplay seems to mostly occurs in extra-striatal circuits. These findings underscore the need for further investigation into the intricate interplay between adenosine and dopamine signaling in striatal and extra-striatal circuits to control central fatigue and other motivational behaviors such as the response to psychostimulants.

## Data Availability

The original contributions presented in the study are included in the article/Supplementary materials, further inquiries can be directed to the corresponding author.
